# Stannous Fluoride Effects on Enamel: A Systematic Review

**DOI:** 10.3390/biomimetics5030041

**Published:** 2020-08-31

**Authors:** Luca Fiorillo, Gabriele Cervino, Alan Scott Herford, Luigi Laino, Marco Cicciù

**Affiliations:** 1 Department of Biomedical and Dental Sciences, Morphological and Functional Images, University of Messina, Policlinico G. Martino, Via Consolare Valeria, 98100 Messina, Italy; gcervino@unime.it (G.C.); mcicciu@unime.it (M.C.); 2 Multidisciplinary Department of Medical-Surgical and Dental Specialties, University of Campania “Luigi Vanvitelli”, 80100 Napoli, Italy; luigi.laino@unicampania.it; 3 Department of Maxillofacial Surgery, Loma Linda University, Loma Linda, CA 92354, USA; aherford@llu.edu

**Keywords:** dentistry, stannous fluoride, toothpaste, enamel, oral health, oral hygiene

## Abstract

In recent years there has been a lot of talk about toothpastes with a particular chemical compound: stannous fluoride (SnF_2_). Its presence is currently still highly controversial, as the latter could have negative health effects. The different companies that produce toothpastes express its dosage in ppm. The purpose of this systematic literature review is to analyze all randomized clinical trials in the literature over the last 10 years and to draw clear results on the function of stannous fluoride, for this purpose the authors performed a Mann–Whitney *U* Test. Materials: The first analysis of the literature produced a number greater than 800 results, subsequently applying the inclusion and exclusion criteria, and following a manual analysis of the results, 26 manuscripts have been obtained. Results: From the results analyzed in this review, it could be shown that stannous fluoride does not present important contraindications, if not those commonly reported for fluorine. A meta-analysis on enamel loss has been conducted, it shows that SnF_2_ products provide better results with a *p* < 0.05 value. Conclusion: This compound could have significant effects in favor of erosion and recalcification of the enamel, on the biofilm formation, gingival inflammation, and in addition, it could be an important aid in the removal of tooth stains and halitosis.

## 1. Introduction

### 1.1. Rationale

Toothpaste is a product aimed at cleaning, maintaining the aesthetics and health of the teeth. Together with the toothbrush it is commonly used to promote oral hygiene. The main functions of this product concern: the removal of food residues from the teeth, the support for the elimination and/or masking of halitosis, the prevention of gum and dental disease, when it also consists of active ingredients such as fluorine or xylitol [1,2]. They represent vehicles through which various active ingredients are applied on the dental surfaces, including antibacterial substances (triclosan, chlorhexidine, cetylpyridinium chloride), bleaching agents (perlite, etc.), desensitizers (amine fluoride), with anti-tartar action (pyrophosphates) or with remineralizing action (stannous fluoride, calcium, phosphates) [3]; remineralizing agents could also be materials that release ions in the mouth (calcium phosphate minerals, bioglass, fluorine salts, etc.) or materials that attach to enamel surface and remineralize it (apatites, amorphous calcium phosphates, etc.). However, it should be noted that they have no action on the removal of the plaque, which occurs exclusively through the mechanical action of the brush [4,5]. Traditionally, toothpaste is presented as a cream (also called toothpaste paste), but it is also marketed in the form of a gel, or with mixed compositions. It is generally extracted from a flexible plastic tube. Its use is almost always carried out by placing a portion of this on a toothbrush (both manual and electric), and through the latter it is spread on the dental arches and between the gums. Chemically it is a sol [6,7].

However, toothpastes or powders did not enter general use until the 19th century. The Greeks, and later the Romans, improved the recipes for toothpaste by adding abrasives such as crushed bones and oyster shells [8]. In the ninth century, the Persian musician Ziryab was known to have invented a type of toothpaste, which he popularized throughout Islamic Spain. The precise ingredients of this preparation are currently unknown, but some sources attest that it was “functional and pleasant to taste”. It is not known whether these first toothpastes were used alone; it is likely that they were rubbed on the teeth with rags, or used together with early toothbrushes, such as tree twigs. In the 16th century, a US and British toothpaste recipe was found that had burnt bread as an ingredient. Another formula from the same period required dragon blood (a resin), cinnamon and burnt alum. In the twentieth century a paste made of hydrogen peroxide and sodium bicarbonate was recommended for use together with the toothbrush. Pre-packaged toothpastes were marketed in the 19th century, but did not exceed the popularity of tooth powder until the First World War. In 1892, Dr. Washington Sheffield of New London developed toothpaste contained in a collapsible tube. This idea was then copied and applied by numerous manufacturers. Fluoride was added to toothpastes in 1914, and this addition was criticized by the American Dental Association (ADA) in 1937. Fluoride toothpastes developed in 1950 received ADA approval, however [9,10,11]. Tooth enamel hydroxyapatite is mainly composed of phosphate ions (PO_4_^3−^) and calcium ions (Ca^2+^). Under normal conditions, there is a stable balance between calcium and phosphate ions in saliva and crystalline hydroxyapatite which makes up 96% of tooth enamel. When the pH falls below a critical level (about 5.5 for enamel and 6.2 for dentin), it causes the tooth mineral (hydroxyapatite) to dissolve in a process called demineralization. When the pH on the tooth surface becomes acidic, the phosphate in oral fluids combines with hydrogen ions (H^+^) to form hydrogen phosphate species (see below). Under these conditions, phosphate is “extracted” from the tooth enamel to restore phosphate levels in saliva and the hydroxyapatite dissolves. When the pH returns to normal, the calcium and phosphate in saliva can recrystallize into hydroxyapatite, remineralizing the enamel. Fluorine can be administered from several different fluorine sources [12,13,14,15]. The three most popular sources of fluoride globally, all of which are accepted by the US FDA as clinically effective, are:

- Stannous fluoride (SnF_2_);
- Sodium fluoride (NaF);
- Sodium monofluorophosphate (Na_2_PFO_3_ or SMFP).

The efficacy of fluoride as a caries preventive agent largely depends on its concentration and availability in oral fluids to influence the demineralization/remineralization balance.

**Figure 1:**
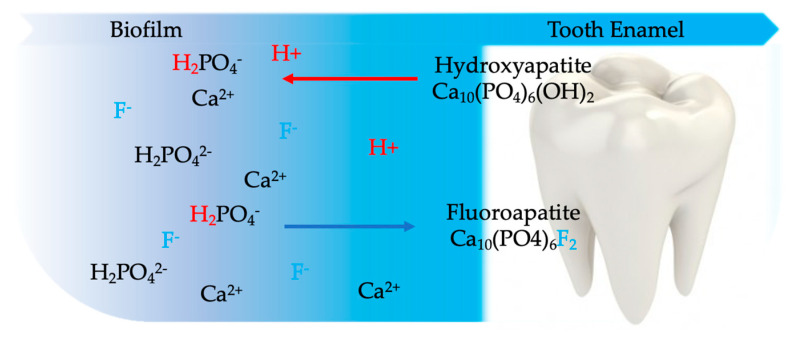
When the pH drops below 5.5, the biofilm fluid becomes undersaturated with phosphate ion and enamel dissolves to restore balance. When fluoride (F^−^) is present, fluorapatite is incorporated into demineralized enamel and subsequent demineralization is inhibited.

Although Stannous Fluoride also has the potential to provide benefits related to the antibacterial properties of the ingredient, this initial formulation provided only a benefit for anticaryosis based on the action of the fluoride. The presence of bioavailable fluoride in the oral fluids (i.e., biofilm and saliva) greatly enhances the crystallization of fluorapatite into tooth structure from calcium and phosphate ions present in saliva. Stannous fluoride adheres to the surface of tooth enamel and forms a protective layer that is able to shield enamel from the effects of erosive acids (Figure 1). Sodium fluoride is a fluoride salt commonly used in dentifrices and oral rinses. Sodium fluoride delivers a highly reactive fluoride ion; therefore, formulating it with a compatible abrasive is critically important for achieving the anticaries benefit. Unlike sodium fluoride, Sodium Monophosphate is not an ionic fluoride salt, but rather a covalently bound compound that requires enzymatic activation by a salivary enzyme (alkaline phosphatase) to release bioavailable fluoride. Because of this lower reactivity, SMFP is compatible with more abrasives than other fluoride sources [16].

Fluoride in various forms is the most popular active ingredient for the prevention of tooth decay in toothpastes [17,18,19]. Although present in small quantities in plants, animals, and in some sources of natural water, as well as having effects on the formation of tooth and bone enamel, it is not considered an essential element of the diet and there are no known signs of pathological deficit in its absence. Sodium fluoride (NaF) is the most common form; some brands use sodium monofluorophosphate (Na_2_PFO_3_) or amine fluoride 297 (C_27_H_60_F_2_N_2_O_3_). The first toothpaste containing biomimetic synthetic hydroxyapatite appeared in Europe as a valid alternative to fluorine for the remineralization and repair of tooth enamel [20,21,22,23]. The function of biomimetic hydroxyapatite is to protect the teeth by creating a new layer around the tooth, hardening the existing enamel which chemically changes to fluorapatite (Ca_5_(PO_4_)_3_F). Biomimetic nano hydroxyapathite has been extensively tested. After nano hydroxyapathite treatment the enamel has been reported to be more mineralized [24] and harder [25] than demineralized enamel. In recent years there has been a lot of talk about a compound, stannous fluoride, its presence in toothpastes is still much debated and the effects are not fully clarified. Stannous fluoride (SnF_2_) is both bacteriostatic and bactericidal and can reduce bacterial growth and control biofilm. There are several active ingredients that are used in remineralizing products: fluorine compounds, calcium phosphates, hydroxyapatite, amorphous forms of calcium phosphate are the most relevant. Fluoride is a mineral that could strengthen teeth and protect them from cavities. It is normally present in drinking water and in some foods (e.g., fish, peanuts and tea), as well as in products for oral hygiene. Useful in the prevention of dental caries, they are local application with the use of fluoride-based toothpastes and mouthwashes, and fluoroprophylaxis treatments during the hygiene sessions in the office. The intake of fluoride, in the form of tablets, can be indicated in the growth phase of the teeth (up to about the age of 12), especially when the drinking water taken daily does not already have an optimal amount of fluoride (from 0, 5 to 1.0 mgF/L) [26]. However, not all variants used by the industry have the same ability to interact with dental tissues and some are definitely not very effective if not as desensitizers. Various formulas have been presented by toothpaste manufacturers to avoid oxidation, including recently one in which the incorporation of zinc phosphate has been proposed [27,28,29].

### 1.2. Objectives

The objectives of this review are to highlight all the clinical features concerning stannous fluoride reported in the literature and eventually its chemical interactions.

- In dental patients, what is the effect of stannous fluoride compositions on oral health compared to other dental healthcare products?

And as secondary outcome:

- On enamel and other hard tooth tissue, what is the effect of stannous fluoride composition on their structure compared to other dental healthcare products?

## 2. Materials and Methods

### 2.1. Protocol and Registration

Well-defined protocols were strictly followed for the preparation of this review. First, a search was conducted in the systematic review databases to highlight similar studies or not. Subsequently the systematic review has been registered on PROSPERO (international database of prospectively registered systematic reviews in health and social care, welfare, public health, education, crime, justice, and international development, where there is a health-related outcome). PROSPERO is produced by CRD and funded by the National Institute for Health Research (NIHR). The number and date of registration (under review) on PROSPERO are as follows: number 176261 on 24/03/2020.

The systematic review was conducted in accordance with the PRISMA (Preferred Reporting Items for Systematic Reviews and Meta-Analyses) statement, all the guidelines were followed (Checklist/Flow diagram), the division into chapters and paragraphs was respected. The analysis of the risk of bias and the setting up of the research, including the drafting of the objective questions of the systematic review, respected the PRISMA criteria, and in particular, in the latter case the PICO (Population/Intervention/Comparison/Outcome) guidelines.

### 2.2. Eligibility Criteria

The full text of all studies of possible relevance was obtained for assessment against the following inclusion criteria:

- Study about stannous fluoride dentifrice/toothpaste/mouth rinse.
- Study of patient side effects of stannous fluoride.
- Study about stannous fluoride chemo-physical interaction.
- Clinical studies on stannous fluoride use and control groups.
- Articles published in the last 10 years.

The applied exclusion criteria for studies were as follows:

- Studies involving subjects with other specific diseases, immunological disorders, oncological patients, osteoporosis, and genetic diseases.
- Not enough information regarding the selected topic.
- No access to the title and abstract in English language or letters, commentary, PhD thesis and editorials.
- Not Randomized Controlled Trial (RCT) studies.

### 2.3. Information Sources

The search strategy has been conducted on different electronic databases. A search on Ovid MEDLINE, PubMed and EMBASE for relevant studies published was carried out. A hand search of the reference lists in the articles retrieved was carried out to highlight any additional publications and to improve the sensitivity.

### 2.4. Search

Search has been conducted using the following keyword “stannous fluoride”. The choice of keywords was intended to collect as much relevant data as possible without relying on electronic means alone to refine the search results. The choice of keywords was made in accordance with the MeSH words (Medical Subject Headings).

### 2.5. Study Selection

Two independent reviewers (L.F. and G.C.) of the University of Messina singularly analyzed the results in order to select inclusion and exclusion criteria. They compared decisions and resolved differences through help of a third expert reviewer (M.C.) For the stage of reviewing of full-text articles, a complete independent dual revision was performed. The results have been compared at the end of the research with a fourth external senior reviewer (A.S.H.). A possible disagreement regarding the inclusion of the studies was discussed among the authors.

### 2.6. Data Collection Process

The first phase of the research consisted of the selection of titles, which allowed us to make a first screening of the manuscript eliminating those not concerning our research. Finally, the full text of all studies was obtained and according to the expected inclusion/exclusion criteria, articles were selected and included in the present review.

### 2.7. Data Items

After the first literature analysis, all article titles were screened to exclude irrelevant publications, case reports, and the non-English language publications. Then, researches were not selected based on data obtained from screening the abstracts. The final stage of screening involved reading the full texts to confirm each study’s eligibility, based on the inclusion and exclusion criteria.

### 2.8. Risk of Bias in Individual Studies

This type of review analyses all the studies in the literature in the last ten years presenting a review of recent data about stannous fluoride clinical effects. Regardless of the results of the studies taken into consideration, the evaluation was carried out on the field of action of the analyses carried out by the studies. Risk of bias analysis has been conducted according to PRISMA guidelines [30,31,32].

### 2.9. Summary Measures

Data were collected from results and arranged in in tables with the following fields (Table 1, Table 2 and Table 3):

Table 1:

- Authors and Year—Authors and year of publication
- Sample size—Information about sample size and sample type
- Groups—Information about number of groups and type of group (each group is separate by “vs.”)
- Time and/or Follow up—Information about timing and follow up of the study
- Main results—Main outcomes and results of the analyzed study
- Statistic results—Statistical results (if performed)

Table 2:

- Main outcome—Classifications of the outcomes obtained by search
- N. of results—Number of obtained results in that outcome set

Table 3:

- Authors and Year—Authors and year of publication
- Sample—Information about sample size and sample type
- Measured at—Obtaining date of the median value
- Mean value—Mean value of groups (SnF_2_ group first vs. other)

### 2.10. Risk of Bias Across Studies

A risk of bias analysis has been conducted and it is shown in Table 2.

### 2.11. Additional Analyses

Having two random, independent samples authors used a Mann–Whitney *U* Test (0.05 significance level) with the null hypothesis that medians of the two samples are identical. Statistical data is shown in Table 4.

## 3. Results

### 3.1. Study Selection

**Figure 2:**
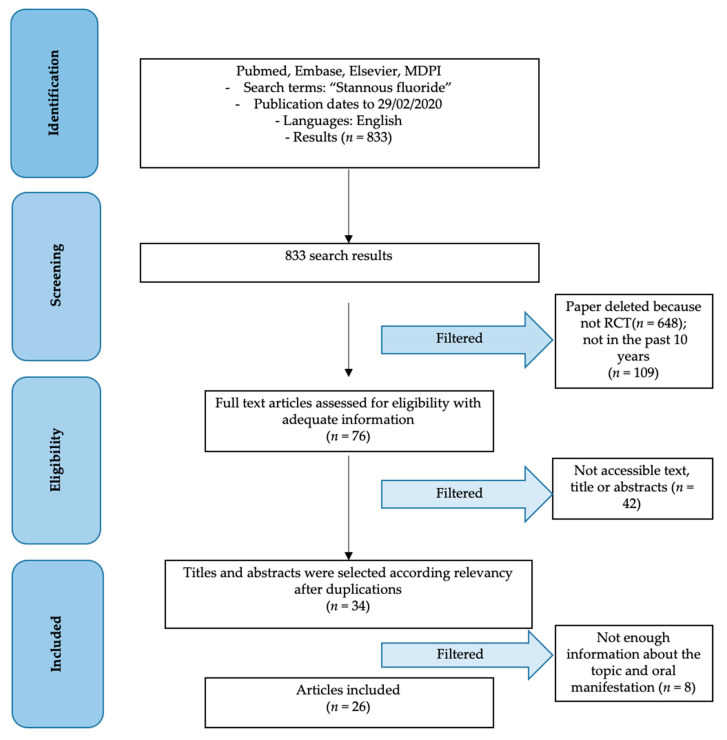
PRISMA flow chart.

From the first research, a total of 833 results were obtained from the scientific databases. Subsequently these results, subjected to screening and application of the inclusion and exclusion criteria, were reduced as follows. Initially only the Randomized Controlled Trial items were selected, obtaining a total of 185 results. Subsequently, only the articles published in the last 10 years were considered, for a total of 76 results remaining. In this review, only the results of the past decade on the effects of stannous fluoride were considered. This aspect, carefully evaluated by the authors, aims to be able to compare in vitro or in vivo study techniques similar to each other, and above all chemical formulations as similar as possible. Therefore, only the accessible and available data articles (34) and subsequently the related ones and with sufficient information to conduct a review were considered (26) (Figure 2).

### 3.2. Study Characteristics

Study characteristics have been shown according to material and methods section and showed in Table 1

### 3.3. Risk of Bias within Studies

Risk of bias analysis for each study has been showed in Table 2 according to Materials and Methods section guidelines.

### 3.4. Results of Individual Studies

West et al. [33] in their double blinded RCT, demonstrated how the use of stannous fluoride could significantly reduce enamel loss compared to 0.24% sodium fluoride dentifrice and a 0.3% triclosan one. Participants wore an intraoral appliance with two polished human enamel samples for six hours a day. They used assigned dentifrice two times a day and swished orange juice for 10 min a day. Seriwatanachai et al. [34] in a double blinded RCT evaluated the use of three different toothpastes. Stabilized SnF_2_ vs. SnF_2_ with zinc lactate vs. a fluoride dentifrice. They provided instruction and randomly assigned different dentifrices to patients. At three visits (0, 3 and 6 months) gingival and plaque index were evaluated. Both SnF_2_ toothpastes provided an index reduction in patients. Luo et al. [35] evaluated sensitivity reduction after an in-office bleaching treatment with different dentifrices after the use of stannous fluoride dentifrices or placebo ones. Tooth sensitivity was evaluated by a Visual Analog Scale (VAS) at day zero, day one, 2, 7, 14 and day 30. Immediately after bleaching the mean VAS values were lower in group 1, with no differences between 2 and 3. Furthermore at days two, seven, 14 and 30 there were no statistical differences. Li et al. [36] in their in vitro blinded study evaluated the use of three different paste/gels on tooth stain reduction. SnF_2_ with 1% zinc paste showed the best results with a 17.5% better reduction at three weeks and a 27.8% reduction at the six-week time. According to Li et al. [36] stabilized Stannous Fluoride toothpaste performs better than non-abrasive toothpaste in this in vitro study; this toothpaste offers a therapeutic dentifrice with good performance in stain prevention and removal. In this case the staining agent consisted of a coffee, wine and tea (1:1:1) combination, used in hot water for 10 min. Ionta et al. [37] in their in vitro study, evaluated the use of three different toothpastes with a fourth placebo group. The calcium silicate, sodium phosphate, and 1450 ppm sodium monofluorophosphate showed better result in enamel wear, after an erosive and abrasive treatment. This study has been conducted on bovine enamel blocks. The investigated dentifrice reduced enamel loss against the acid challenge but had no effect against the acid and brushing challenge. In another double-blinded study of Hu et al. [38], it could be obtained how SnF_2_ toothpaste provides better results than common fluoride dentifrices. SnF_2_ toothpaste helps to reduce the inflammation index and to gain better plaque control. Haraszthy et al. [39] evaluated the effect of fluoride toothpaste on bacteria reduction, with promising results. Hagenflield et al. [40] in their study, evaluated differences in the microbiome after the use of two anti-adhesive and antibacterial toothpastes during periodontal therapy on a mild and moderate periodontitis population. They did not notice any differences in microbiome diversity. Creeth et al. [41] investigated in a short-term clinical study, how a stannous fluoride toothpaste could reduce DH compared to brushing with a conventional toothpaste after single use. This toothpaste, effectively, reduced DH on evaporative and tactile stimuli after a three-day treatment. It provided a significant Schiff sensitivity score and a tactile score reduction. Zero et al. [42] showed that all 1400–1450 ppm F dentifrices provide better remineralization than placebo, except for the SnF_2_ group. According to West et al. [43] there is a high grade of tubule occlusion in the toothpaste containing 0.454% stannous fluoride and in the fluoride toothpaste containing 0.76% sodium monofluorophosphate brushed dentine samples. There are no statistical differences between the first and second group of study. According to Frese et al. [44] an increase of caries-free surface between groups was observed. They highlighted a decrease of caries superficialis and media too. This four-year RCT was conducted on a population of athletes. According to authors, it could be shown how the odds of developing caries media on a new surface was significantly lower. In their RCT, West et al. [45] made 33 subjects wear an appliance with human enamel. They subdivided the sample into two groups, a SnF_2_ group and a triclosan group. The group 1 provided statistically significant better results about enamel loss from day 10. A previous study of West et al. [46] provided valuable in vitro and in situ results about SnF_2_ toothpaste. Their human enamel samples showed a greater protection against dental erosion if SnF_2_ toothpaste was used instead of a sodium monofluorophosphate (SMFP)/arginine dentifrice. Marchetti et al. [47] evaluated plaque regrowth on a three-day mouthwash program. The differences between the groups were statistically significant, in particular, the use of an alcohol-free essential oil provided the worst results on plaque formation. The best result was provided by the use of a CHX mouthwash twice daily, followed by SnF_2_ with zinc mouthwashes. Geidel et al. [48] obtained significant results about API and OHI reduction in all groups. However, herbal toothpaste resulted in significantly lower API and OHI. Hove et al. [49] in their study, evaluated the effect of four different toothpastes on human molars, mounted on mouth appliances and worn by eight volunteers for nine days. To mimic the gastric effect, the specimens were etched for two minutes a day (extra-orally). The SnF_2_ specimens showed significantly lower enamel wear than the control group (fluoride free toothpaste, group 1) and group 4 with the same toothpaste of control and an additional 0.4% SnF_2_ solution for two minutes. This solution fully protected enamel surface, better than the 0.4% SnF_2_ toothpaste (group 2) and 0.454% SnF_2_ toothpaste (group 3). Bellamy et al. [50] demonstrated how a SnF_2_ toothpaste can protect from enamel loss and that there is no differences between group 1 and 3. Another Bellamy et al. study [51] evaluated the plaque inhibition effect of SnF_2_/NaF dentifrice, they demonstrated how in a 17-day period this dentifrice could provide significant results on plaque inhibition. Stenhagen et al. [52] evaluated enamel resistance to erosive/abrasive with the use of different agents. Sodium fluoride, stannous fluoride, titanium tetrafluoride was compared to a control group mouth rinse. Human enamel specimens with one amalgam filling were used, and worn for nine days by volunteers. According to authors SnF_2_ and TiF(4) had better results. According to Jentsch et al. [53] there are significant differences between different oral rinses against plaque regrowth. They compared the use of essential oils, amine/stannous fluoride and chlorhexidine digluconate. CHX had better results on plaque thickness and cocci and bacilli counts already after 24 h. West et al. [54], in their randomized crossover trials, compared stannous fluoride and essential oil mouth rinse. They used two different experimental stannous fluoride compositions and evaluated tooth and tongue staining. These formulations obtained good results on teeth staining vs. essential oils or water. Fine et al. [55] evaluated the effect of three dentifrices formulations. They collected samples from four sites, plaque, saliva, tongue and buccal mucosa, and evaluated six microbial types, anaerobes, Streptococci, Actinomyces, hydrogen-sulphide (H_2_S)-producing bacteria, Fusobacteria and Veillonella. The use of sodium fluoride/triclosan/copolymer (TCN/C) demonstrated reduction on microbiota. SnF_2_/SHMP showed significant reduction compared with the NaF group. Huysmans et al. [56] demonstrated on worn human enamel samples, how SnF_2_ toothpastes could reduce enamel erosion. Authors conducted a scanning electron microscope (SEM) analysis on enamel samples. Group 1 reduced erosive wear by 34% and group 2 by 26% with a significant difference to the control group (group 3). Wigger-Alberti et al. [57] evaluated the efficacy of different mouthrinses formulation in reducing oral malodor. They compared 250 ppm F(-)amine fluoride/stannous fluoride (ASF), 0.2% zinc lactate, oral malodour counteractives versus chlorhexidine mouthrinses (group two 0.05% CHX, 0.05% cetylpyridinium chloride, 0.14% zinc lactate; group three: mouthrinse III (0.12% CHX) and with a control group (tap water). ASF mouthrinse showed a better effect on discoloration and organoleptic ratings. All data about groups of the cited studies in this paragraph have been shown in Table 1.

### 3.5. Additional Analysis

Through the use of a Mann–Whitney *U* Test, it is possible to evaluate statistical differences between obtained data. Unfortunately, these data are inhomogeneous, and only an enamel wear loss statistical analysis has been performed (Table 4).

On eight studies, one study has been removed due to bovine enamel blocks use and another one for incomplete data. It is possible to demonstrate that the Critical value of *U* at *p* < 0.05 is 11. Therefore, the result is significant at *p* < 0.05. The *p*-value is 0.04648. There is a significant difference between SnF_2_ use (toothpaste or mouthwashes) versus control groups on enamel wear loss. The result is significant at *p* < 0.05.

### 3.6. Risk of Bias Across Studies

The studies taken into consideration in this review, being current, are few. The results obtained from this review may be skewed by an incorrect assessment within the studies and across the studies. The results were analyzed according to the methods listed in the previous chapter. Unfortunately, it was not possible to carry out a statistical analysis on the few data present and compare them to each other due to a lack of homogeneity of the measurements. However, none of the studies analyzed showed missing data or selective reporting. Surely it would have been an advantage to have a double blind when studying the samples.

It is also necessary to report that some studies could be influenced by an unreported conflict of interest, also given the important commercial nature of the study. The evaluated studies have investigated animal models, succeeding in demonstrating SnF_2_ products effects. The risk of bias is defined as low. It is preferable to use simple approaches for assessing validity that can be fully reported.

## 4. Discussion

### 4.1. Summary of Evidence

The literature articles provide useful information to clarify the functions and effects of this chemical compound in toothpastes. A discussion of the results, reporting here a critique of the conclusions of the individual scientific articles has been carried out. The use of different compounds in toothpastes has been much debated, mainly due to the presence of chemicals that could have more or less serious health effects. For example, orange juice and other citrus juices are perceived with an unpleasant taste after using toothpaste since the chemical interaction between the stannous fluoride present in the toothpaste and the acetic acid contained in the juice causes an alteration of the flavor. Sodium lauryl sulphate alters the taste perception, generally increasing the bitter taste, breaking down the phospholipids that inhibit the receptors of this taste; it is thought to also inhibit sweet taste receptors. On the other hand, the apple is known to have a pleasant flavor after the use of toothpaste. It is still an unsolved problem to distinguish whether the cause of the alteration of the orange juice taste is due to stannous fluoride or sodium lauryl sulfate; it is thought that the added aroma (often menthol) could also take part in the alteration of the perception of taste, when it is binding in the lingual receptors of the cold [58]. Fluoride is used in most toothpastes as an active ingredient. Many controversies arose as a result of the daily use of this substance. Fluoride toothpaste seems to cause damage to health: in fact, the intake of quantities of fluorine greater than 2 mg per day causes fluorosis. If a large amount of toothpaste is ingested, a poison control center should be contacted immediately. In the USA, a wording is required on the tubes of toothpaste, inviting to contact a doctor or a control center, in case of ingestion of an excessive amount of toothpaste, and therefore of fluorine [59,60,61]. The effectiveness of fluorine is questioned by some chemists and scholars from all over the world, who appeal above all to an increasing number of studies that would highlight the toxicity of fluorine salts, capable of causing, with minimal overdoses, of fluorosis; ruining bones and teeth, and causing nervous system problems and cognitive deficits [62]. However, the articles cited refer to the toxicity of fluorine food supplements, without prejudice to the usefulness of the fluorine contained in toothpastes which instead performs an effective protective activity. In this context, it is worth underlining that the availability of efficient antiseptic systems, without particular contraindications, can be indicated for pregnant women, as it limits the phenomenon of premature births and therefore of underweight babies that recent studies hypothesize to be related to the presence of gingivitis, pathologies that causes the release into the circulation of factors, such as metalloproteases, capable of interacting with the hormonal systems and consequently stimulating early uterine contractions. There are no allergic reactions reported in results. Other scholars counter these criticisms, stating instead that the advantages of using fluorine as an aid in the mineralization of dental enamel are also demonstrated by natural observations in populations residing in neighboring areas in the presence of waters rich in fluorine ions, in which it is present a low incidence of caries. It is also noteworthy that the risk of using fluoride is quite low and indeed the use of toothpaste with a high fluoride content (1350–1500 ppm) is recommended for all ages (although smaller volumes are used for children small; a “stain” of toothpaste up to 3 years) [63,64,65]. There are several fluoride-free toothpaste options available on the market for those who choose not to use this element. There are commercially available toothpastes based on delicate washing substances (sodium cocoglucoside tartrate, white clay) and soothing plant extracts for gums and periodontal health (extract of myrrh, chamomile, krameria triandra), peppermint oil, strawberry plant extract. Some natural toothpastes use carboxymethylchitosan, present in the exoskeleton of crustaceans, as an anticaries active ingredient [66]. This homoglycan is able to bring the pH values of the mouth closer to neutrality by neutralizing lactic acid; it also has antimicrobial properties. Stannous fluoride showed good antibacterial properties in animal studies too. It is worth mentioning, even if briefly, some results that were excluded from the research phase because they did not meet the criteria (older than 10 years). A study by Yu et al. [67] evaluated the potential to reduce enamel and dentin erosion with a single application of stannous chloride-containing fluoride solution. This was assessed on both enamels covered by the enamel film and dentin under acidic conditions in vitro. According to the authors the staus chloride-containing fluoride solution reduced calcium loss of enamel and dentine to up to 6 and 3.5 min, respectively. Even a single application therefore showed positive effects. Wang et al. [68] investigated the fluoride-releasing ability of a tooth separator consisting of elastomer and fluoride. The enamel area contacting with the separator and its surrounding area showed lower mineral loss compared to control with no tooth separator application. Stookey et al. [69] evaluated the anticaries effectiveness of a low-dose sodium fluoride dentifrice in 2004. In this clinical trial authors indicated that while no difference in caries increments was observed between the low-NaF and control groups with high-NaF and the SnF_2_-HMP and control groups. Orbak et al. [70] examined the effect of an electro-ionizing toothbrush with stannous fluoride in the treatment of dentin hypersensitivity. Authors showed how an ionizing brush may be an effective tool for treatment of dentin hypersensitivity in the post-periodontal surgery.

According to Seltzer et al. [71] stannous fluoride products (BacDerm; Emerald 3 Enterprises Inc., Camdenton, MO, USA) can significantly reduce a bacterial skin infection. This product showed significant reduction on hair coat, odour, pruritus on dogs. Toothpastes containing amine fluoride and stannous fluoride perform remineralizing action by forming a precipitate of calcium fluoride, adhering to the tooth surface, capable of subsequently releasing fluorine particles (Figure 1). Stannous fluoride has excellent antiplaque and antibacterial potential. In fact, it is able to penetrate the bacterial membrane, accumulating inside the cell and inhibiting the activity of the bacterial enzymes involved in the production of acids from sugar. Thanks to its natural acidity, in aqueous solutions it causes a reduction in the pH inside the bacterial cells, also preventing the activity of other enzymes [72,73,74,75,76].

In addition, it appears that stannous fluoride is able to block interactions between the bacteria themselves and between the bacteria and the surface of the teeth. In this way it prevents bacteria from accumulating on the tooth surfaces. However, the use of stannous fluoride as an active ingredient in oral hygiene products presents some difficulties. The divalent ions of which it is composed are very reactive: in aqueous environments and in the presence of oxygen they dissociate within a few hours to form a white precipitate; the ions oxidize to form tetravalent tin ions (Sn_4_ +). Unfortunately, these reactions, which occur in the presence of water and oxygen, also lead to the inactivation of stannous fluoride, reducing its antibacterial effects. The imperfections on the enamel surface are filled with micrometric aggregates of apatites nanocrystals forming a biomimetic protective coating. This also repairs dentin and reduces dentinal sensitivity.

#### 4.1.1. Hard Tissue Effects of Stannous Fluoride Compounds

According to West et al. [33] stannous fluoride dentifrice shows a greater erosion protection relative to the NaF/triclosan compositions. Ionta et al. [37], in their conducted study on bovine enamel block, showed how calcium silicate, sodium phosphate, and 1450 ppm sodium monofluorophosphate dentifrice could reduce enamel loss against the acid challenge. Hu et al. demonstrated how a SnF_2_ dentifrice improves clinical outcomes and how in the six month-period time evaluation, it provided an improvement in all evaluated indexes. West et al. [45] demonstrated how a stabilized SnF_2_ dentifrice provides superior protection against tooth enamel surface loss compared to NaF/triclosan dentifrice. The triclosan/copolymer technology is compatible with the fluoride to which it can be associated in toothpaste formulations. In another study, West et al. [46] confirmed the superiority of stabilized SnF_2_ dentifrice for protecting human teeth from erosion. According to Hove et al. [49] 0.4% SnF_2_ solution mouthwash, after a fluoride-free toothpaste brushing, provided better results on enamel protection, better than SnF_2_ toothpastes too. SnF_2_ toothpaste could protect enamel from erosion according to Bellamy et al. [50]. SnF_2_ toothpaste provided better results than the NaF dentifrice or control group (water). Stenhagen et al. [52] demonstrated how SnF_2_ mouth rinse could reduce enamel erosion if compared to TiF_4_ and NaF in human enamel samples. Huysmans et al. [56] with their SEM analysis concluded that SnF_2_ toothpaste could reduce erosive tooth wear in situ. West et al. [43] concluded that there are no differences in tubule occlusion capability between toothpaste containing 0.454% stannous fluoride and fluoride toothpaste containing 0.76% sodium monofluorophosphate. However, following the acid challenge, there was a statistically significantly greater degree of occlusion in the stannous fluoride toothpaste.

#### 4.1.2. Biological and Plaque Effects of Stannous Fluoride Compounds

Haraszthy et al. [39] concluded that stannous fluoride dentifrice provides a bacteria reduction 12 h after brushing, and microbial reduction continues four hours later, with promising result on long term use. According to Hagenfield et al. [40] the use of a toothpaste with anti-adhesive zinc-substituted carbonated hydroxyapatite did not provide changes on microbial composition versus anti-adhesive amine fluoride/stannous fluoride, but authors evaluated microbial composition after oral hygiene and periodontal therapy. Marchetti et al. [47] in their randomized crossover clinical trial, proved how, CHX mouthwashes provide better results against plaque regrowth compared to alcohol-free essential oil or SnF_2_ mouthwashes. Geidel et al. [48] in their study, concluded that after 24 weeks of controlled study, the herbal toothpaste, compared to a stannous fluoride toothpaste, was as good as the control toothpaste, with no side effect. In terms of periodontal health, the herbal toothpaste could be a suitable alternative to conventional dentifrices. Bellamy et al.’s [51] population showed less plaque coverage with the use of SnF_2_ toothpaste at a 17-day period than a fluoride dentifrice. Jentsch et al. [53] demonstrated how chlorhexidine digluconate mouth rinses could reduce plaque thickness and counts of cocci and bacilli after use, it obtained better results if compared to essential oils and amine/stannous fluoride. These last two groups did not differ between us. Fine et al. [55] demonstrated that TCN/C dentifrice formulation consistently reduced for a range of microorganisms in diverse oral sites in comparison with the NaF, or the SnF_2_/SHMP dentifrice formulations as seen 12 h after brushing. Seriwatanachai et al. [34] showed how SnF_2_ dentifrice could help patients against plaque formation and a gingival index reduction.

#### 4.1.3. Oral Health Related Quality of Life Effects of Stannous Fluoride Compounds

According to Li et al. [36] stabilized stannous fluoride toothpaste performs better on tooth staining removal than regular fluoride toothpastes. Lorenz et al. [49] performed a RCT, in healthy dental students, with no mechanical oral hygiene and eight daily rinses with mouth rinse and black tea. They evaluated the effect of three different rinses on five groups: AmF/SnF_2_ rinse (3 groups), essential oil rinse and water. All rinses led to tooth and tongue staining, with statistical differences in tooth staining between groups. According to obtained results, group number 3 had promising potential on less tooth discoloration than other AmF/SnF_2_ rinses. Lorenz et al. [49] proved that one of the experimental AmF/SnF_2_ rinses leads to less staining than others. West et al. [54] concluded that stannous fluoride mouth rinses could provide good results on teeth staining formation. According to Wigger-Alberti et al. [57], ASF products showed better results than CHX products on discoloration and oral malodour (organoleptic score and volatile sulfur compounds). Luo et al. [35], comparing stannous fluoride group with potassium nitrate and placebo, affirmed that the use of potassium nitrate could alleviate tooth sensitivity during and after in-office bleaching. Creeth et al. [41] demonstrated how brushing with an experimental anhydrous 0.454% SnF_2_ polyphosphate toothpaste reduced DH with a single use and better in three days use.

## 5. Limitations

The main limitation of this study is given by the fact that it is not possible to couple the single results and carry out a univocal statistical analysis, as the results come in the single results evaluated using different parameters. Data from the last ten years were considered in order not to create discrepancies between types of experimentation and between different chemical formulations of toothpaste. Only English-language articles were considered and this may be a limitation.

## 6. Conclusions

Although it was not possible to perform a meta-analysis due to the incomparable results, it is evident how much the individual studies support the use of compounds with stannous fluoride for oral hygiene. This compound has demonstrated different functions and characteristics, in the absence of reported contraindications. The contraindications in fact, are those for fluorine, from overdose. The stannous fluoride has been shown to have positive effects both in terms of plaque formation, tooth stains and gingival inflammation. The antimicrobial effects of staus fluoride are often overcome by other components such as chlorhexidine. As for the other outcomes, the results appear to be promising versus sodium fluoride, herbal toothpaste or triclosan products. A meta-analysis on enamel loss has been conducted, it shows that SnF_2_ provides better results with *p* < 0.05 value on enamel wear loss than in control groups. Furthermore, it appears to have excellent results with respect to the remineralization of the enamel, even in the presence of demineralized areas, or in the occlusion of the exposed dentinal tubules.

## Figures and Tables

**Table 1:** Results of individual studies table. This table shows results according to paragraph summary measures.

Authors and Year	Sample Size	Groups	Time and/or Follow up	Main Results	Statistic Results
West et al. [33] 2019	36	2 Groups: 0.454% stannous fluoride dentifrice vs. market dentifrice NaF/triclosan (0.24% sodium fluoride and 0.3% triclosan)	10 days trial	Stannous fluoride dentifrice demonstrated 93.5% less enamel loss than control	*p <* 0.001
Seriwatanachai et al. [34] 2019	135	3 Groups: Stabilized SnF_2_ dentifrice vs. SnF_2_ with zinc lactate dentifrice vs. a fluoride dentifrice	6 months	Both SnF_2_ dentifrice showed a statistically significant reduction of gingival inflammation and plaque. With no statistical differences between themselves	*p <* 0.001
Luo et al. [35] 2019	150	3 Groups: (48) Potassium nitrate vs. (45) stannous fluoride vs. (46) placebo	30 days	Authors demonstrated how Potassium nitrate toothpaste could reduce sensitivity after an in-office bleaching treatment, with no differences between stannous fluoride and placebo.	*p <* 0.05
Li et al. [36] 2019	18 bovine enamel sample	3 Groups: 0.454% SnF_2_ and 1% zinc phosphate vs. Crest Pro-Health Whitening Power vs. non-abrasive SnF_2_ gel	6 weeks	In this in vitro study SnF_2_ and 1% zinc paste performed better results than competitor and non-abrasive gel. It showed a better tooth stain reduction with no adverse effect.	*p <* 0.01 at 3 weeks
Ionta et al. [37] 2019	256 bovine enamel sample	4 Groups: calcium silicate, sodium phosphate, and 1450 ppm sodium monofluorophosphate vs. dentifrice with 3500 ppm stannous chloride, 700 ppm amine fluoride, and 700 ppm sodium fluoride vs. conventional dentifrice, with 1450 ppm sodium monofluorophosphate vs. control (deionized water)	20 days	The group 1 promoted less enamel loss than water (group 4) but it did not differ from group 2 or 3. But group 1 dentifrice promoted a higher wear after erosion than other groups.	*p <* 0.05
Hu et al. [38] 2019	100	2 Groups: SnF_2_ dentifrice vs. fluoride dentifrice	6 months	Both groups had a significant reduction in gingival inflammation and a plaque control improvement. SnF_2_ dentifrice showed a reduction of all indexed compared to control dentifrice	*p <* 0.001
Haraszthy et al. [39] 2019	129	2 Groups: Stannous fluoride toothpaste vs. sodium monofluorophosphate toothpaste	8 weeks	Stannous fluoride group showed a greater reduction of bacteria. From 14% at time zero to 27% at 4 weeks, and 41% at 8-week time.	*p <* 0.05
Hagenfield et al. [40] 2019	41	2 Groups: anti-adhesive zinc-substituted carbonated hydroxyapatite (HA) vs. with antimicrobial and anti-adhesive amine fluoride/stannous fluoride (AmF/SnF_2_)	12 weeks	There were no differences between groups in microbiome changes.	*p* > 0.05
Creeth et al. [41] 2019	656	2 Groups: (329) experimental anhydrous 0.454% SnF_2_/polyphosphate toothpaste vs. (327) toothpaste containing 0.76% sodium monofluorophosphate	3 days	Experimental toothpaste reduced dentine hypersensitivity (DH) after 3 days treatment better than test group.	*p <* 0.0001
Zero et al. [42] 2018	168	4 Groups: sodium fluoride (NaF)/Carb/silica, NaF/silica, NaF + monofluorophosphate (MFP)/chalk vs. NaF/Carb/silica, NaF + MFP/dical, amine fluoride (AmF)/silica vs. NaF/Carb/silica, NaF + stannous fluoride (SnF_2_)/silica/hexametaphosphate (HMP) vs. Placebo (0 ppm F) and/or dose-response controls (675 ppm F as NaF [675F-NaF]) ±Carb	14 days	All 1400–1450 ppm F dentifrices except NaF + SnF_2_/silica/HMP provided significantly greater lesion remineralization than Placebo. Carb addition did not alter fluoride efficacy.	*p* *<* 0.0001
West et al. [43] 2018	21 samples	3 Groups: toothpaste containing 0.454% stannous fluoride vs. Control fluoride toothpaste containing 0.76% sodium monofluorophosphate vs. mineral water	10 days	After 4 days of treatment the degree of tubule occlusion increased in the dentine samples in the groups 1 and 2 than in water.	*p <* 0.01
Frese et al. [44] 2018	54	2 Groups: special stannous fluoride-containing [(AmF)/NaF/SnCl ] mouth rinse (500 ppm F^−^, 800 ppm Sn^2+^), 1 × 30 s and a special toothpaste containing NaF/Sn^2+^ and the biopolymer chitosan (elmex EROSIONSSCHUTZ, CPGABA GmbH, Hamburg, Germany) vs. fluoridated toothpaste (1500 ppm)	4 years	Two groups showed similar caries prevalence. There was a decrease of caries superficialis and media.	\
West et al. [45] 2017	33 human enamel sample	2 Groups: 0.454% SnF_2_/0.077% NaF vs. 0.32% NaF/0.3% triclosan.	15 days	SnF_2_ group provided a reduction of enamel loss at day 10 and again at day 15.	*p <* 0.0001
West et al. [46] 2017	33	2 Groups: SnF_2_ + 0.77% sodium fluoride (NaF) vs. sodium monofluorophosphate/arginine dentifrice	10 day	Group 1 provided better enamel protection against erosive acid challenge than group 2	*p <* 0.0001
Marchetti et al. [47] 2017	20	3 Groups: Alcohol free essential oil mouthwash vs. Amine fluoride/stannous fluoride with zinc lactate mouthwash vs. chlorhexidine (CHX) mouthwash	3 days	Group 1 showed better results on plaque regrowth compared to alcohol-free essential oil mouthwash. But there was a less impact if compared to CHX.	*p <* 0.001
Geidel et al. [48] 2017	76	3 Groups: Herbal toothpaste vs. triclosan/copolymer toothpaste vs. amine/stannous fluoride toothpaste	24 weeks	Approximal plaque index (API) and Oral hygiene index (OHI) changed in all groups with a significantly lower API e OHI in group 1. Sulcus bleeding index (SBI) was improved in all groups after 12 weeks. Bleeding on Probing (BoP) was unchanged.	*p* = 0.001
Lorenz et al. [49] 2015	28	5 Groups: amine fluoride/stannous fluoride (AmF/SnF_2_), 250 ppm F^−^; low concentration of film-forming agents; low concentration of humectants vs. amine fluoride/stannous fluoride, 250 ppm F^−^; low concentration of film-forming agents, high concentration of humectants vs. amine fluoride/stannous fluoride, 250 ppm F^−^; high concentration of film-forming agents; high concentration of humectants vs. Phenolic/essential oil mouth rinse vs. Volvic Still Water, Danone Waters	10 days	All mouth rinses led to tooth and tongue staining, statistically significant differences existed between groups 1, 3, 4 and 5 on tooth staining	\
Hove et al. [50] 2014	64 human teeth sample	4 Groups: Fluoride-free toothpaste vs. toothpaste 0.4% SnF_2_ vs. toothpaste 0.454% SnF_2_ vs. fluoride free toothpaste and a 0.4% SnF_2_ solution (1000 ppm F)	9 days	The SnF_2_ groups showed significantly lower enamel wear than the group 1	*p <* 0.05
Bellamy et al. [51] 2014	12	3 Groups: sodium fluoride dentifrice vs. Stannous fluoride dentifrice vs. water	15 days	Enamel loss was significantly lower for treatment in group 2 versus 1 or 3	*p <* 0.005
Bellamy et al. [52] 2014	27	2 Groups: SnF_2_/sodium fluoride (NaF) dentifrice vs. anticavity dentifrice	17 days	Group 1 showed better results on 17 days usage period, it demonstrated a statistically significant a lower mean plaque area at each timepoint.	*p <* 0.0001
Stenhagen et al. [53] 2013	16 molars sample	4 Groups: NaF vs. SnF_2_ vs. TiF_4_ vs. control	9 days	The mean surface loss in the NaF, SnF_2_ and TiF_4_ groups was significantly lower than in the control group	*p <* 0.05
Jentsch et al. [54] 2013	24	3 Groups: Essential oil mouth rinse vs. amine/stannous fluoride mouth rinse vs. chlorhexidine digluconate 0.12% mouth rinse	96h	The counts of cocci and bacilli and plaque thickness are statistically different only in chlorhexidine digluconate 0.12% group, with positive results	*p* ≤ 0.05
West et al. [55] 2012	20	4 Groups: AmF/SnF_2_ mouthrinse 250 ppm, F— 430 ppm Sn vs. AmF/SnF_2_—mouthrinse 250 ppm, F—430 ppm Sn vs. essential oil vs. water	4 days	Rinse 2 produced less stain than rinse 1, but the difference was not significant. Rinse 2 produced significantly more stain than rinse 3 and 4. For tongue staining, rinse 2 produced significantly more staining than 4 but not 1 or 3.	*p* < 0.05
Fine et al. [56] 2012	35	3 Groups: Sodium fluoride/triclosan/copolymer dentifrice vs. Stannous fluoride/sodium hexametaphosphate/zinc lactate dentifrice (SnF_2_/SHMP) vs. sodium fluoride dentifrice	13 days	Group 1 demonstrated significant reduction on plaque compared to other groups.	*p <* 0.01
Huysmans et al. [57] 2011	20 enamel samples	3 Groups: SnF_2_ toothpaste (1050 ppm fluoride from stan- nous fluoride and 350 ppm from amine fluoride) vs. SnF_2_ toothpaste (containing 1100 ppm fluoride from stannous fluoride and 350 ppm from sodium fluoride) vs. sodium fluoride toothpaste	5 days	SnF_2_ toothpastes significantly reduced erosive wear.	*p <* 0.05
Wigger-Alberti et al. [58] 2010	174	4 Groups: Amine fluoride/stannous fluoride 0.2% zinc lactate mouthrinse + malodour counteractives vs. 0.05% CHX, 0.05% cetylpyridinium chloride, 0.14% zinc lactate mouthrinse vs. (0.12% CHX mouthrinse vs. tap water.	21 days	Group 1 showed efficacy to teeth discoloration, a significant reduction of organoleptxic ratings and volatile sulfur compounds was achieved after single application and after days 7 and 21.	*p <* 0.001

**Table 2:** Risk of bias analysis according to PRISMA, as specified in paragraph risk of bias (“+”: low risk of bias; “-“: high risk of bias; “?”: unclear risk of bias).

Author	Random Sequence Generation (Selection Bias)	Allocation Concealment (Selection Bias)	Blinding of Participants and Personnel (Performance Bias)	Blinding of Outcome Assessment (Detection Bias)	Incomplete Outcome Data (Attrition Bias)	Selective Reporting (Reporting Bias)
West et al. [33] 2019	+	+	+	+	+	+
Seriwatanachai et al. [34] 2019	+	+	+	+	+	+
Luo et al. [35] 2019	+	+	-	-	+	+
Li et al. [36] 2019	+	+	+	+	+	+
Ionta et al. [37] 2019	+	-	+	-	+	+
Hu et al. [38] 2019	+	+	+	-	+	+
Haraszthy et al. [39] 2019	+	+	-	-	+	+
Hagenfield et al. [40] 2019	+	+	+	+	+	+
Creeth et al. [41] 2019	-	-	+	+	+	+
Zero et al. [42] 2018	+	+	-	-	+	+
West et al. [43] 2018	+	+	+	-	+	+
Frese et al. [44] 2018	+	+	-	-	+	+
West et al. [45] 2017	+	+	+	+	+	+
West et al. [46] 2017	+	+	+	+	+	+
Marchetti et al. [47] 2017	+	-	+	+	+	+
Geidel et al. [48] 2017	-	+	-	-	+	+
Lorenz et al. [49] 2015	+	-	+	-	+	+
Hove et al. [50] 2014	-	-	-	-	+	+
Bellamy et al. [51] 2014	+	-	-	-	+	+
Bellamy et al. [52] 2014	+	-	+	+	+	+
Stenhagen et al. [53] 2013	-	-	-	-	+	+
Jentsch et al. [54] 2013	-	-	-	-	+	+
West et al. [55] 2012	+	-	+	-	+	+
Fine et al. [56] 2012	+	+	+	+	+	+
Huysmans et al. [57] 2011	+	+	-	-	-	+
Wigger-Alberti et al. [58] 2010	+	-	+	+	+	+

**Table 3:** Main outcome table as specified in summary measures paragraph.

Main Outcome	No. of Results
Enamel loss reduction [17,21,29,30,33,34,36,40]	9
Bacteria and others microorganisms reduction [23,24,31,32,35,37,39]	7
tooth stain reduction [20,38,49]	4
Gingival inflammation reduction [18,22,32]	2
Dentinal hypersensitivity reduction [19,25]	2
Carious lesion remineralization [26,28]	2
Dentin tubule occlusion [27]	1
organoleptic ratings and volatile sulfur compounds [41]	1

**Table 4:** Obtained data and meta-analysis on enamel wear loss.

Author and Year	Sample	Measured at	Mean Value
West et al. 2019 [33]	36 Intraoral appliances with human enamel sample	Day 10	0.097 µm vs. 1.495 µm
Ionta et al. 2019 [37]	256 Bovine enamel blocks	Day 5	4.8 ± 2.5 µm vs. 4.8 ± 1.4 µm
West et al. 2017 [43]	33 Intraoral appliances with human enamel sample	Day 15	1.60 µm vs. 5.03 µm
West et al. 2017 [44]	33 Human enamel specimens	Day 15	5.75 µm vs. 23.75 µm
Hove et al. 2014 [50]	16 Intraoral appliances with human molar	Day 9	14.5 ± 9.3 µm and 33.3 ± 7.4 µm and 0.4 ± 1.3 µm vs. 29.2 ± 10.5 µm
Bellamy et al. 2014 [51]	12 intraoral appliances with human enamel sample	Day 15	2.03 µm vs. 15.53 µm
Stenhagen et al. 2013 [53]	16 intraoral appliances with molar sample	Day 9	1.8 ± 1.9 µm vs. 26.3 ± 4.7 µm vs. 3.1 ± 4.8
Huysmans et al. 2011 [57]	20 intraoral appliances with human enamel sample	Day 5	data not clear or incomplete
